# Biometric Variations in High Myopia Associated with Different Underlying Ocular and Genetic Conditions

**DOI:** 10.1016/j.xops.2022.100236

**Published:** 2022-10-25

**Authors:** Hashem H. Ghoraba, Cassie A. Ludwig, Darius M. Moshfeghi

**Affiliations:** Byers Eye Institute, Stanford University, Palo Alto, California

**Keywords:** High myopia, Biometry, Axial length, Retinopathy of prematurity, Stickler syndrome, AC, anterior chamber, ACD, anterior chamber depth, AK, average keratometric readings, AXL, axial length, FEVR, familial exudative vitreoretinopathy, IHM, isolated high myopia, LT, lens thickness, OCTA, OCT angiography, ROP, retinopathy of prematurity

## Abstract

**Purpose:**

To report different biometric measurements in high myopia associated with different underlying ocular and genetic conditions.

**Design:**

Retrospective study.

**Subjects:**

Patients with high myopia.

**Methods:**

We searched the Stanford Research Repository tool to identify patients with the diagnosis of high myopia who were seen by a single provider at Byers Eye Institute at Stanford from January 2019 to March 2022. We performed a chart review and included eyes that had high myopia and ocular biometric measurements at any time point after January 2019. We divided our cohort into 5 different groups: (1) isolated high myopia (IHM) (control group); (2) retinopathy of prematurity (ROP); (3) familial exudative vitreoretinopathy; (4) Marfan syndrome; and (5) Stickler syndrome.

**Main Outcome Measures:**

Biometric measurements.

**Results:**

A total of 246 patients (432 eyes) were included as follows: 202 patients (359 eyes) in the IHM group, 17 patients (27 eyes) in the ROP group, 7 patients (12 eyes) in the familial exudative vitreoretinopathy group, 8 patients (14 eyes) in the Marfan group, and 12 patients (20 eyes) in the Stickler group. The ROP group showed significantly shorter axial lengths, shallower anterior chambers, and thicker lenses compared with the IHM group. The Marfan group showed significantly flatter corneas and thicker lenses compared with the IHM group. The Stickler group showed significantly longer axial lengths compared with the IHM group.

**Conclusions:**

High myopia is associated with variable biometric measurements according to underlying ocular or genetic conditions. Retinopathy of prematurity-associated high myopia is primarily lenticular, while Stickler syndrome-associated high myopia is axial. Marfan syndrome-associated high myopia is derived from both axial and lenticular mechanisms.

It is estimated that by 2050, 50% of the world population will be myopic and that as high as 10% will have high myopia.^1^ There is a consensus that a myopia epidemic is currently ongoing.^1^, ^2^, ^3^ High myopia is commonly defined by a spherical equivalent refractive error of ≤ −5.00 or ≤ −6.00 diopters or axial length ≥ 26–26.5 mm.^4^^,^^5^ Pathologic and high myopia are occasionally used interchangeably but “pathologic myopia” should refer to myopia associated with structural complications, especially in the posterior segment.^4^^,^^5^ Nonetheless, high myopia is commonly associated with pathologic myopia.^5^

Myopia varies across different ethnicities regarding prevalence, age of onset, and progression pattern.^6^ Myopia is also related to both genetic and environmental factors, although the recent epidemic is largely attributed to environmental influences.^1^^,^^4^^,^^6^^,^^7^ Myopia has been classified according to macular findings.^8^ Additionally, our group has previously noted that myopia may be divided into isolated high myopia (IHM), anterior myopia, posterior myopia (e.g., myopic maculopathy), and combined anterior and posterior.^9^^,^^10^ Therefore, myopia is currently addressed by structural macular changes as well as location-based anatomic changes.

Different hereditary and acquired disorders have been associated with myopia including Stickler syndrome,^11^ Marfan syndrome,^12^ retinopathy of prematurity (ROP),^13^ and familial exudative vitreoretinopathy (FEVR).^14^ The pathophysiology of myopia entails longer axial lengths, steeper corneal curvatures, lens changes, or a mixture of all these factors. Myopia secondary to excessive axial elongation is named axial myopia, while myopia associated with increased refractive power, whether corneal or lenticular, is named refractive myopia.^4^

In this study, we began with the hypothesis that the underlying hereditary and acquired ocular causes of high myopia may result in different biometric measurements. We sought to investigate if etiology impacted findings.

## Methods

### Study Setting and Subjects

This was a retrospective, observational study in which we searched the Stanford Research Repository tool to identify patients with the diagnosis of progressive high (degenerative) myopia, using International Classification of Diseases codes H44.20, 21, 22, and 23, who were seen by a single provider (D.M.) at Byers Eye Institute at Stanford from January 2019 to March 2022. We performed a chart review and included eyes that had a confirmed diagnosis of high myopia, defined as spherical equivalent refractive error of ≤ −6.0 diopters or axial length ≥ 26.5 mm, and had ocular biometric measurements at any time point after January 2019. We excluded eyes that did not meet the criteria of high myopia, eyes with no biometric measurements, eyes with scleral buckling procedures, and eyes with a history of trauma or infection.

The study was conducted in compliance with the Declaration of Helsinki, the United States Code of Federal Regulations Title 21, and the Harmonized Tripartite Guidelines for Good Clinical Practice (1996). Stanford University Institutional Review Board approved the study under protocol number 36544, and an informed consent waiver was obtained as the charts of enrolled patients were retrospectively reviewed.

### Data Collection and Outcomes

We collected demographic data, underlying ocular and genetic conditions, manifest refraction measurements, biometric measurements, and optical coherence tomography (OCT) findings. We divided our cohort into 5 different groups according to the presence or absence of the most common underlying ocular or genetic conditions in our cohort namely (1) IHM (control group); (2) ROP; (3) FEVR; (4) Marfan syndrome; and (5) Stickler syndrome. The IHM group included eyes with no known underlying ocular or genetic conditions.

Biometric data were collected using IOL Master (software version V.7.5, Carl Zeiss Meditec AG) including axial length (AXL), average keratometric readings (AK), anterior chamber depth (ACD), lens thickness (LT), and white to white. After controlling for age and sex, we compared different biometric measurements across the 5 different groups as well as individually between the control group and each of the other groups. Refraction, LT, and ACD were not considered in eyes with cataract greater than grade 1 nuclear sclerosis, pseudophakia, or aphakia. Refraction and AK were not considered in eyes with history of refractive surgery.

Macular OCT images were graded into eyes with and without fovea plana, which was defined as the absence of a normal foveal pit on OCT. The relationship between fovea plana and axial length was explored.

We used the Stanford REDCap for the collection and management of data. The Stanford REDCap platform (http://redcap.stanford.edu) is developed and operated by Stanford Medicine Research IT team. The REDCap platform services at Stanford are subsidized by (a) Stanford School of Medicine Research Office and (b) the National Center for Research Resources and the National Center for Advancing Translational Sciences, National Institutes of Health, through grant UL1 TR001085.

### Statistical Analysis

Descriptive statistics were calculated for the variables of interest. Continuous variables were expressed in mean and standard deviation. Analyses were run using linear mixed-effects models. Different groups and the variable of interest were included in all models as a categorical fixed effect. Bonferroni correction was performed to adjust for multiple comparisons. To control for age and sex, their values were also included as fixed effects on all models. Measures were sometimes taken on both eyes, therefore, samples from the same patient were not independent. To avoid averaging measures or eliminating data, we included “Patient” as a random intercept effect. We ran the models using restricted maximum likelihood and verified model quality and assumptions by testing and visually evaluating for deviations of dispersion and residuals homogeneity of fixed and random effects.

Data analysis was performed using RStudio software (version 1.3.1093).

## Results

**Figure 1:**
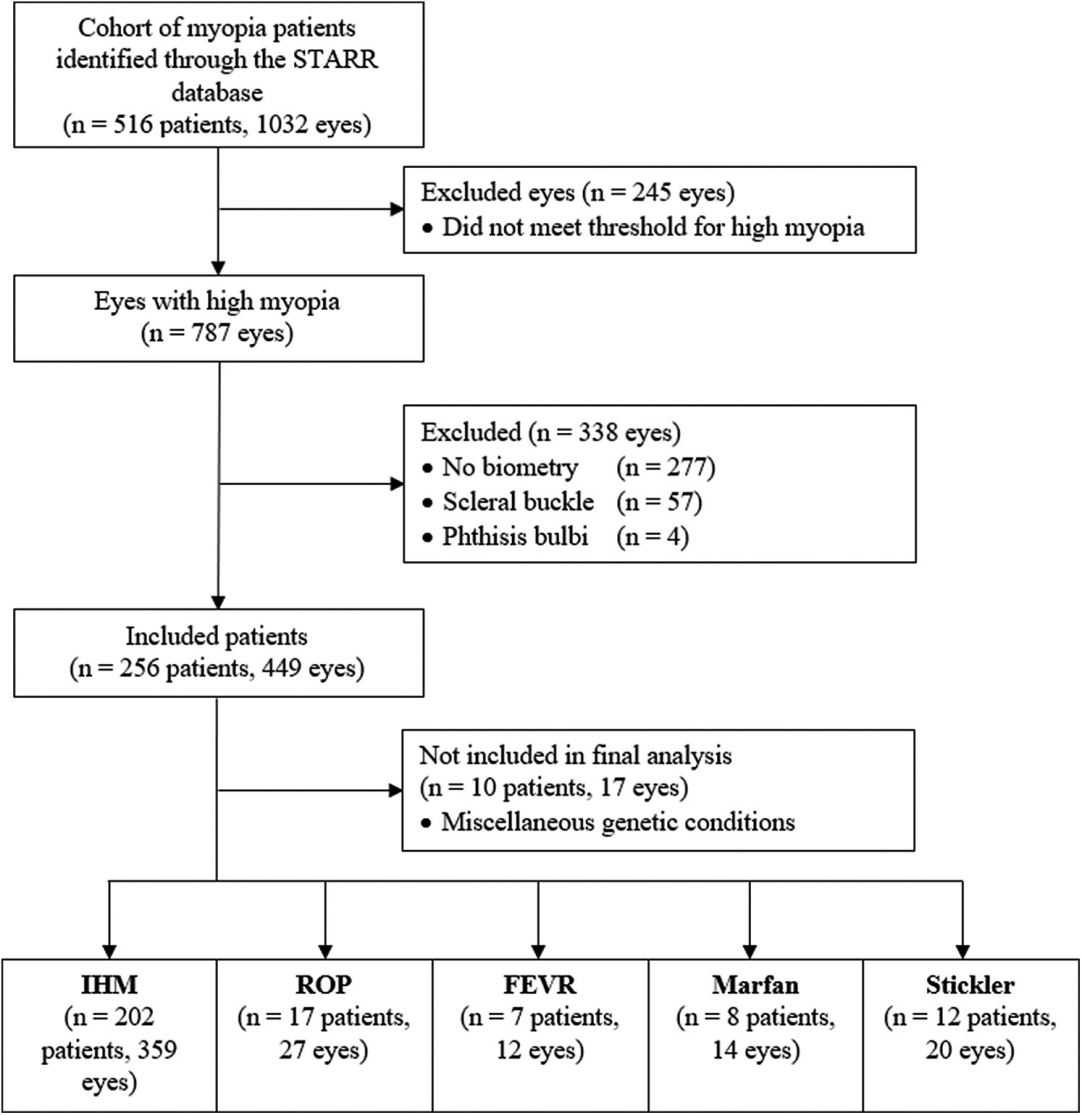
Consolidated standards of reporting trials cohort selection diagram. IHM = isolated high myopia; FEVR = familial exudative vitreoretinopathy; ROP = retinopathy of prematurity.

A total of 516 patients (1032 eyes) were initially identified through the Stanford Research Repository tool. A total of 256 patients (449 eyes) met the inclusion criteria. Ten patients (17 eyes) were further excluded as they did not fit any of the 5 groups and had miscellaneous underlying ocular or genetic conditions including ocular albinism, choroideremia, X-linked retinoschisis, and homocystinuria. A total of 246 patients (432 eyes) were included in the final analysis (Figure 1).

### Biometric and Refractive Measurements

**Figure 2:**
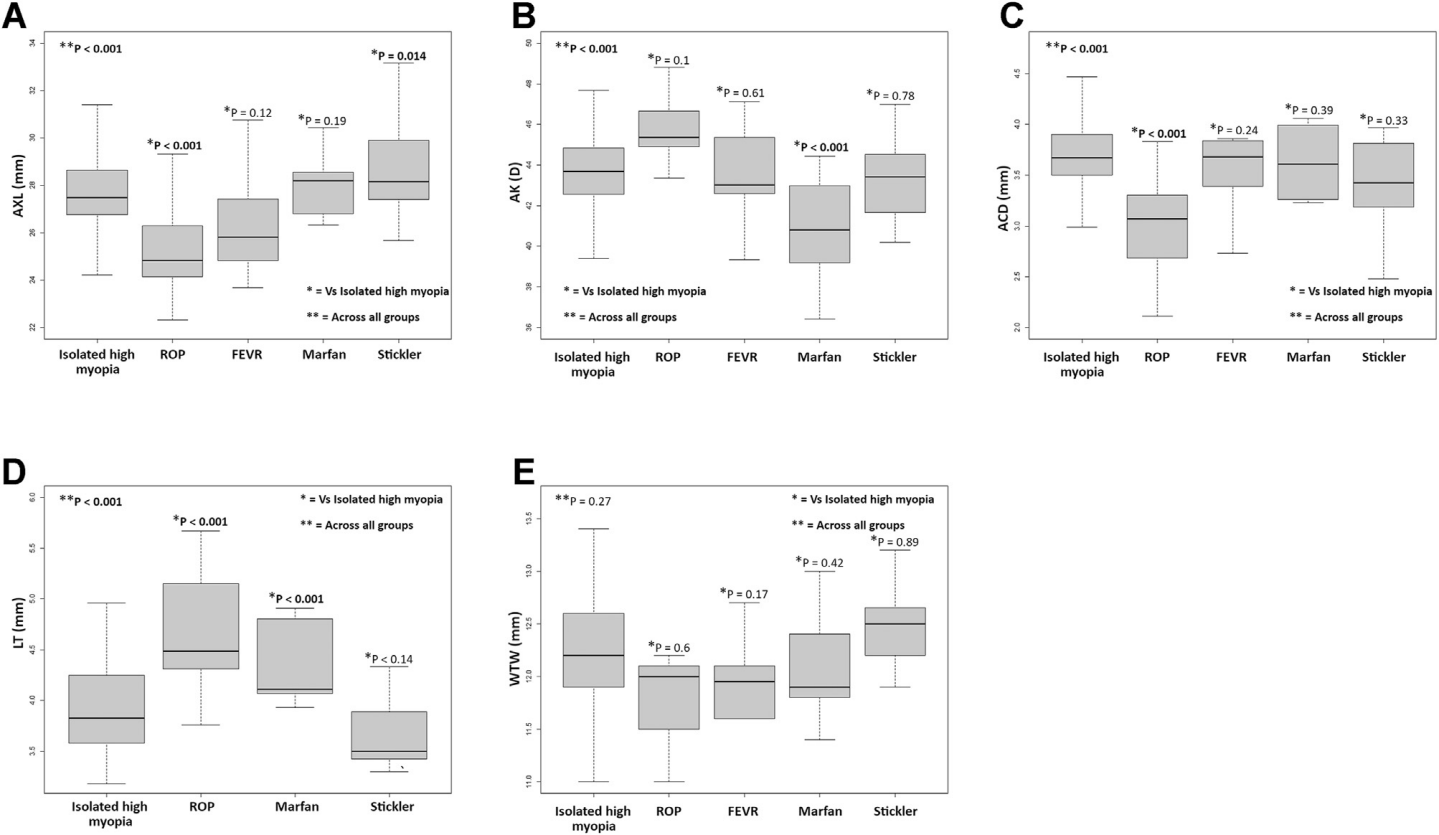
Boxplots illustrating different biometric measurements across the different groups. **A,** Axial length (AXL) measurements. **B,** Average keratometric readings (AK). **C,** Anterior chamber depth (ACD). **D,** Lens thickness (LT). **E,** White to white (WTW). FEVR = familial exudative vitreoretinopathy. *P* values represent adjusted *P* values.

Table 1 summarizes baseline criteria and biometric and refractive measurements of the 5 groups. Figure 2 shows variations in biometric measurements of the 5 groups. AXL, AK, ACD, LT, and spherical equivalent refractive errors showed statistically significant differences across the different groups. Eyes associated with Stickler syndrome had the longest AXL (mean 28.8 mm), while eyes with ROP were associated with the shortest AXL (mean 25.3). Eyes with ROP had the highest AK readings (mean 45.7 D), while eyes associated with Marfan syndrome were associated with flat corneas (mean 40.7 D). Eyes with ROP were associated with significantly shallow ACD (mean 3 mm) compared with the other groups. Eyes with ROP and eyes associated with Marfan syndrome had thicker lenses (means of 4.6 and 4.4 mm, respectively) compared with eyes with Stickler syndrome and IHM. On average, eyes associated with Stickler syndrome had the highest spherical equivalent refractive errors (mean −14 D), and eyes with IHM had the lowest refractive errors (mean −10.6). White-to-white measurements were slightly different between the 5 groups, but the differences were not statistically significant after adjustment for age, sex, and AXL.

**Table 1:** Demographic and Biometric Measurements of Eyes with High Myopia by Different Groups

	IHM	ROP	FEVR	Marfan Syndrome	Stickler Syndrome	*P* Value∗
Number of patients (eyes)	202 (359)	17 (27)	7 (12)	8 (14)	12 (20)	
Age (mean ± SD)	50.9 ± 21.4 years	21.2 ± 11.3 years	18 ± 11.4 years	18.1 ± 6.9 years	15.1 ± 8.9 years	
Sex (% female)	56%	23.5%	28.5%	87.5%	33.3%	
Refraction (mean ± SD)†	−10.6 ± 4.1 D	−13.1 ± 4.8 D	−10.7 ± 2.9 D	−13.6 ± 3.1 D	−14 ± 5.7 D	**<0.001**#
AXL (mean ± SD)	27.9 ± 1.7 mm	25.3 ± 1.9 mm	26.5 ± 2.6 mm	28.1 ± 2.8 mm	28.8 ± 2.1 mm	**<0.001**#
AK (mean ± SD)‡	43.6 ± 2.1 D	45.7 ± 1.9 D	43.4 ± 2.4 D	40.7 ± 2.4 D	43.3 ± 2 D	**<0.001**#
ACD (mean ± SD)§	3.7 ± 0.33 mm	3 ± 0.5 mm	3.5 ± 0.4 mm	3.6 ± 0.4 mm	3.4 ± 0.5 mm	**<0.001**#
LT (mean ± SD)‖	3.9 ± 0.4 mm	4.6 ± 0.6 mm	N/A¶	4.4 ± 0.4 mm	3.7 ± 0.4 mm	**<0.001**#
WTW (mean ± SD)	12.2 ± 0.5 mm	11.8 ± 0.5 mm	11.9 ± 0.4 mm	12.1 ± 0.5 mm	12.4 ± 0.5 mm	0.27∗∗

ACD = anterior chamber depth; AK = average keratometric readings; AXL = axial length; D = diopter; FEVR = familial exudative vitreoretinopathy; IHM = isolated high myopia; LT = lens thickness; N/A = not applicable; ROP = retinopathy of prematurity; SD = standard deviation; WTW = white to white. Bold values indicate statistical significance.

∗ Differences across 5 groups.

† Refraction was only considered in phakic eyes with no cataract or history of refractive surgery.

‡ AK was not considered in eyes with a history of refractive surgery.

§ ACD was not considered in eyes with cataracts, pseudophakia, or aphakia.

‖ LT was not considered in eyes with cataracts, pseudophakia, or aphakia.

¶ Missing LT data in the FEVR group.

# Linear mixed-effects model adjusted for age and sex.

∗∗ Linear mixed-effects model adjusted for age, sex, and AXL.

### Differences in Biometric Measurements Between Eyes with IHM and Each of the Other Groups

Table 2 and Figure 2 show the results of direct comparisons between the IHM group and each of the other groups.

**Table 2:** Differences in Biometric Measurements Between Eyes With Isolated Myopia and the Other Groups

	Biometric Measurement	Parameter Value in Comparative Group	Parameter Value in Isolated Myopia	Chi-Square	*P* Value∗
ROP vs. IHM	**AXL**	**25.3 ± 1.9 mm**	**27.9 ± 1.7 mm**	**269.9**	**< 0.001**†
	AK‡	45.7 ± 1.9 D	43.6 ± 2.1 D	2.7	0.10†
	**ACD**§	**3 ± 0.5 mm**	**3.7 ± 0.33 mm**	**32.0**	**< 0.001**†
	**LT**‖	**4.6 ± 0.6 mm**	**3.9 ± 0.4 mm**	**27.8**	**< 0.001**†
	WTW	11.8 ± 0.5 mm	12.2 ± 0.5 mm	0.3	0.60‡
FEVR vs. IHM	AXL	26.5 ± 2.6 mm	27.9 ± 1.7 mm	2.41	0.12
	AK‡	43.4 ± 2.4 D	43.6 ± 2.1 D	0.27	0.61
	ACD§	3.5 ± 0.4 mm	3.7 ± 0.33 mm	1.36	0.24
	LT	N/A¶	3.9 ± 0.4 mm	-	-
	WTW	11.9 ± 0.4 mm	12.2 ± 0.5 mm	1.87	0.17
Marfan vs. IHM	AXL	28.1 ± 2.8 mm	27.9 ± 1.7 mm	1.69	0.19
	**AK**‡	**40.7 ± 2.4 D**	**43.6 ± 2.1 D**	**25.16**	**< 0.001**
	ACD§	3.6 ± 0.4 mm	3.7 ± 0.33 mm	0.72	0.39
	**LT**‖	**4.4 ± 0.4 mm**	**3.9 ± 0.4 mm**	**21.77**	**< 0.001**
	WTW	12.1 ± 0.5 mm	12.2 ± 0.5 mm	0.65	0.42
Stickler vs. IHM	**AXL**	**28.8 ± 2.1 mm**	**27.9 ± 1.7 mm**	**6.08**	**0.014**
	AK‡	43.3 ± 2 D	43.6 ± 2.1 D	0.08	0.78
	ACD§	3.4 ± 0.5 mm	3.7 ± 0.33 mm	1.2	0.33
	LT‖	3.7 ± 0.4 mm	3.9 ± 0.4 mm	2.23	0.14
	WTW	12.4 ± 0.5 mm	12.2 ± 0.5 mm	0.02	0.89

ACD = anterior chamber depth; AK = average keratometric readings; AXL = axial length; FEVR = familial exudative vitreoretinopathy; IHM = isolated high myopia; LT = lens thickness; ROP = retinopathy of prematurity; vs. = versus; WTW = white to white. Boldface indicates parameters and *P* values with statistical significance.

∗ Linear mixed-effects model adjusted for age and sex.

† Adjusted for age, sex, and refraction.

‡ AK was not considered in eyes with a history of refractive surgery.

§ ACD was not considered in eyes with cataracts, pseudophakia, or aphakia.

‖ LT was not considered in eyes with cataracts, pseudophakia, or aphakia.

¶ Missing LT data in the FEVR group.

### ROP versus IHM

The ROP group showed statistically significant shorter AXL, shallower ACD, and thicker lenses when compared with the IHM group (*P* < 0.001 for all 3 parameters). Although AK and white-to-white measurements were higher and lower, respectively, in the ROP group, the differences were not statistically significant after controlling for age, sex, and refraction (Table 2 and Figure 2).

### FEVR versus IHM

In the univariate analysis and before adjustment for age and sex, the FEVR group had significantly shorter AXL measurements when compared with the IHM group (*P* = 0.004). However, after adjustment, the difference was not statistically significant. None of the other measurements was statistically significant in the univariate analysis (Table 2 and Figure 2).

### Marfan versus IHM

The Marfan group showed statistically significant flatter corneas and thicker lenses when compared with the IHM group (*P* < 0.001 for both parameters). None of the other measurements was statistically different between the 2 groups (Table 2 and Figure 2).

### Stickler versus IHM

The Stickler group showed statistically significant longer AXL when compared with the IHM group (*P* **=** 0.014). None of the other measurements was statistically different between the 2 groups (Table 2 and Figure 2).

### Relationship Between Fovea Plana on OCT and AXL

Eighteen eyes were graded with fovea plana on OCT. Most of these eyes were associated with ROP (94.4%, n = 17) and 1 eye was associated with Stickler syndrome. In the ROP group, the mean (standard deviation) of AXL in eyes with (n = 17) and without (n = 10) fovea plana was 24.8 ± 1.2 mm and 26.1 ± 2.6 mm, respectively, and the difference was not statistically significant (*P* = 0.18).

## Discussion

The results of our manuscript suggest that eyes with high myopia have significantly variable biometric measurements according to the underlying ocular or genetic condition. We have found that eyes with high myopia in association with ROP are more likely to have shorter AXL, shallower anterior chamber (AC), and thicker lens when compared with eyes with IHM. Similarly, eyes with high myopia associated with Marfan syndrome have flatter corneas but thicker lenses compared with eyes with IHM. Eyes with Stickler syndrome have higher refractive errors and AXLs than eyes with IHM.

### ROP

In our ROP group, despite the fact that refractive errors were significantly higher compared with the IHM, AXL measurements were significantly shorter. In addition, LT in our ROP group was significantly higher than in the IHM group. This supports the conclusion that high myopia in association with ROP is refractive rather than axial. Furthermore, after controlling for refraction, LT remained significantly higher than IHM, but the AK readings were not. This supports that myopia in ROP is mainly lens induced. This is further supported by the significantly shallow AC measurements in ROP eyes compared with IHM and all other groups, which could be explained by disproportionately large lenses in shorter eyes.

Our results are similar to multiple other studies which noted that in ROP, AXL and ACD measurements are shorter and lenses are thicker than in normal eyes.^15^, ^16^, ^17^, ^18^, ^19^, ^20^ Garcia-Valenzuela and Kaufman^15^ noted that LT and power were strongly associated with high myopia in ROP. They also noted that AXL measurements were significantly shorter. Regarding the corneal power, they concluded that the corneal power contributed in a small but significant proportion to the degree of myopia in ROP. Overall, the authors suggested that ROP-associated myopia is mainly lenticular. This finding is very similar to our study which also suggested that corneal power is increased in ROP compared with IHM but is not as significant as the lens in contribution to myopia.

Choi et al^18^ followed up eyes in premature infants for 6 years and classified the eyes according to the presence of ROP and the degree of myopia. They concluded that eyes with higher degrees of ROP had higher degrees of myopia which they attributed to shallower ACs, thicker lenses, and longer AXLs. They also found no relationship between the keratometric values and the degree of myopia. Their conclusions were similar to, but slightly different from, our study. First, they found no contribution of the cornea to myopia in ROP, which we noted to be present, but to a small degree. Second, they found that eyes with ROP and high myopia had higher AXLs, which we did not demonstrate in our study. We attribute the differences, namely minor corneal contribution, and short AXL, to the fact that we compared our ROP group to eyes with IHM, and they compared their eyes with ROP-associated myopia to eyes with no myopia but with a history of prematurity or ROP. It is likely that ROP eyes with myopia have slightly longer AXL than ROP eyes without myopia. However, when eyes with ROP-associated myopia are compared with IHM, the AXL will be significantly shorter. As for the corneal measurements, it is possible that the increase in corneal power, which was noted in our study as well as previous studies such as Garcia-Valenzuela and Kaufman,^15^ be attributed to the prematurity itself, and hence, the absence of difference if the comparison was performed between premature groups.

Kent et al^17^ proposed that the previously mentioned differences in ROP eyes are secondary to arrest in anterior segment growth. They attributed shallow AC to anterior displacement of the lens together with increased LT. It is not clear if there is a true lens displacement, or if it is just a disproportionately large lens in a shorter eye that is causing the shallow AC. Regardless of the cause of shallow AC, decreased corneal-lens distance increases the refractive power of the eye, and multiple studies, including ours, have shown that it is associated with ROP-associated myopia.

### FEVR

Our FEVR group showed significantly shorter AXL measurements when compared with IHM before controlling for age. Following control for age and sex, the correlation became statistically insignificant. We would be careful to draw hard conclusions regarding our FEVR group given the relatively low number of eyes and patients. Qi et al^21^ reported their findings in a small cohort of eyes with FEVR and high myopia and found that the degree of myopia in their cohort was not associated with long AXL but rather with lenticular changes, similar to the ROP case.

### Marfan

In our Marfan group, AK readings were significantly lower than all of the other groups, including IHM. Our findings are supported by multiple previous studies which showed that Marfan syndrome is associated with flat corneas.^22^, ^23^, ^24^, ^25^ Our study has found that eyes with Marfan syndrome and high myopia not only still have flat corneas when compared with IHM but also when compared with other underlying conditions such as Stickler syndrome and ROP.

Our Marfan group also had increased LT when compared with IHM. Gehle et al^25^ have found that eyes in Marfan syndrome have thicker lenses when compared with control eyes but did not investigate the relationship with high myopic eyes. We also found no difference in AXL between the Marfan group and the IHM group. We suggest that high myopia in Marfan syndrome is derived from both axial (supported by elongated AXL similar to IHM) as well as lenticular (supported by increased LT) mechanisms. The combination of both mechanisms seems to overcome the flat cornea and explain the high degree of myopia in the Marfan group.

### Stickler

Our Stickler group showed a statistically significant increase in AXL compared with IHM. Moreover, AXL measurements and refractive errors of the Stickler group were the highest in our cohort. To the best of our knowledge, this is the first time to report that Stickler syndrome is associated with higher AXL measurements when compared with high myopia due to different underlying conditions.

Foveal hypoplasia, or fovea plana as described by Marmor et al,^26^ is defined as the absence of a normal foveal pit. Chen et al^27^ have found that FEVR was more associated with foveal hypoplasia and showed higher degrees of myopia and longer AXL when compared with ROP and premature infants. Some authors have suggested that fovea plana can be associated with shorter or longer AXL measurements.^28^ In our cohort, none of the eyes in the FEVR group showed fovea plana and most of the eyes with fovea plana were noticed in the ROP group. In addition, we could not find a relationship between fovea plana and AXL measurements.

We recognize that our study has several limitations. Initial patient selection was based on International Classification of Diseases coding. We performed a meticulous chart review to account for this error. Given that all of our cohort was seen by a single retina specialist in a single tertiary center, there is a risk for selection and referral biases. The relatively small number of patients in some of our groups, namely FEVR and Marfan, could have led to missed findings and may limit the generalizability of our conclusions.

## Conclusion

High myopia is associated with variable biometric measurements according to different underlying ocular or genetic conditions. Retinopathy of prematurity-associated high myopia is primarily lenticular, while Stickler syndrome–associated high myopia is axial. Marfan syndrome–associated high myopia is derived from both axial and lenticular mechanisms.
